# Diet and the evolution of *ADH7* across seven orders of mammals

**DOI:** 10.1098/rsos.230451

**Published:** 2023-07-12

**Authors:** Swellan L. Pinto, Mareike C. Janiak, Gwen Dutyschaever, Marília A. S. Barros, Adrian Guadamuz Chavarria, Maria Pia Martin, Fred Y. Y. Tuh, Carmen Soto Valverde, Lisa M. Sims, Robert M. R. Barclay, Konstans Wells, Nathaniel J. Dominy, Daniel M. A. Pessoa, Matthew A. Carrigan, Amanda D. Melin

**Affiliations:** ^1^ Department of Anthropology and Archaeology, University of Calgary, 2500 University Dr NW, Calgary, Alberta, Canada T2N 1N4; ^2^ Alberta Children's Hospital Research Institute, Calgary, Alberta, Canada; ^3^ BE Bioinsight & Ecoa, Nilo Peçanha 730, conj. 505, Porto Alegre, Rio Grande do Sul, Brazil; ^4^ Área de Conservación Guanacaste, Guanacaste, Costa Rica; ^5^ Kids Saving the Rainforest Wildlife Rescue Center, 60601 Quepos, Costa Rica; ^6^ Sabah Parks, 88100 Kota Kinabalu, Sabah, Malaysia; ^7^ Department of Biological Sciences, University of Calgary, Alberta, Canada T2N 1N4; ^8^ Department of Biosciences, Swansea University, Singleton Park, Swansea SA2 8PP, UK; ^9^ Department of Anthropology, Dartmouth College, Hanover, NH 03755, USA; ^10^ Department of Physiology and Behavior, Federal University of Rio Grande do Norte, Natal, Brazil; ^11^ BioTork, Gainesville, FL, USA; ^12^ Department of Anatomy & Physiology, College of Central Florida, Ocala, FL, USA; ^13^ Department of Medical Genetics, University of Calgary, Alberta, Canada

**Keywords:** ethanol metabolism, alcohol dehydrogenase, dietary adaptation, comparative genetics, mammals

## Abstract

Dietary variation within and across species drives the eco-evolutionary responsiveness of genes necessary to metabolize nutrients and other components. Recent evidence from humans and other mammals suggests that sugar-rich diets of floral nectar and ripe fruit have favoured mutations in, and functional preservation of, the *ADH7* gene, which encodes the ADH class 4 enzyme responsible for metabolizing ethanol. Here we interrogate a large, comparative dataset of *ADH7* gene sequence variation, including that underlying the amino acid residue located at the key site (294) that regulates the affinity of ADH7 for ethanol. Our analyses span 171 mammal species, including 59 newly sequenced. We report extensive variation, especially among frugivorous and nectarivorous bats, with potential for functional impact. We also report widespread variation in the retention and probable pseudogenization of *ADH7*. However, we find little statistical evidence of an overarching impact of dietary behaviour on putative *ADH7* function or presence of derived alleles at site 294 across mammals, which suggests that the evolution of *ADH7* is shaped by complex factors. Our study reports extensive new diversity in a gene of longstanding ecological interest, offers new sources of variation to be explored in functional assays in future study, and advances our understanding of the processes of molecular evolution.

## 1. Introduction

Carbohydrates in the form of sugars are the primary nutritive reward of floral nectars and fruit [1,2]. This essential truth extends to a wide range of pollinators and frugivores, but also naturally occurring yeasts, which metabolize sugars for their own nutritive needs. Ethanol is a natural by-product of this process, but the extent to which it deters or attracts competing consumers is a matter of debate [3–5]. Dietary ethanol can have toxic or nutritive effects on consumers according to their digestive abilities or the concentrations ingested, which are highly variable. For instance, some nectars and exudates have ethanol concentrations exceeding 3.0% (v/v), whereas those of primate-edible fruits range from 0.5% to 8.1% [6–13]. Higher levels may adversely impact the normal functioning and fitness of consumers. Furthermore, chronic exposure to ethanol has considerable and diverse health consequences, although the impact of low ethanol concentrations and volumes is less certain [14]. Overall, the extent of ethanol exposure is expected to exert a selective pressure on the catabolic efficiency of metabolic pathways [13,15].

The genes underlying this process are an inviting subject of study, in part because genetic variation among humans and other mammals points to functional variation in relevant enzymes [15,16]. In humans, ethanol is primarily metabolized via the conversion of ethanol to acetaldehyde via alcohol dehydrogenase (ADH) enzymes, and later converted to acetic acid via aldehyde dehydrogenases (ALDHs) [17]. Due to its expression in the upper gastrointestinal tract, ADH class IV (encoded by the *ADH7* gene, also referred to as *ADH4* in some studies; [17]) plays a key role in the first-pass metabolism of ethanol [18]. In particular, an amino acid change from alanine to valine at site 294 of this gene, which probably evolved in the last common ancestor of humans and African apes, led to a 40-fold increase in enzymatic activity for ethanol [16,19,20]. This change has been hypothesized to be an adaptation to increased dietary ethanol exposure, possibly in response to increased terrestriality and higher exposure to fallen, fermenting fruits on the ground [16]. Interestingly, the distantly related aye-aye lemur (*Daubentonia madagascariensis*), an omnivorous species well known to favour floral nectars of the traveller's tree (*Ravenala madagascariensis*), acquired this mutation independently [16,21]. Preference for ethanolic solutions (3–5%) in an experimental study provides first experimental evidence of a role for ethanol in food choice of aye-ayes [22]. Moreover, previously reported loss-function mutations of *ADH7* in lineages of mammals with herbivorous or carnivorous diets [15] suggest that nectar- and fruit-heavy diets may exert selective pressure on the evolution of this gene. However, a study focused on a sufficiently large sample of frugivorous and/or nectarivorous species for comparative analyses is needed to test this hypothesis more directly.

**Figure 1.**
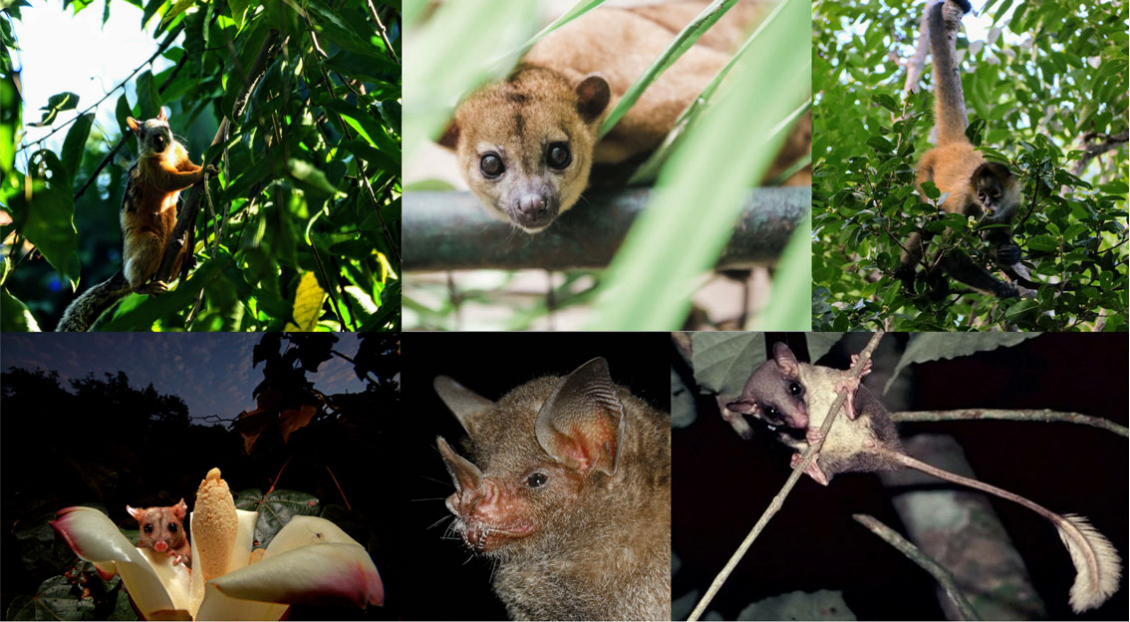
Frugivory and/or nectarivory has evolved repeatedly in mammalian evolution. Images are examples of frugivorous/ nectarivorous species across six orders of mammals: (top left) Rodentia, variegated squirrel (*Sciurus variegatoides*), image by Amanda Melin; (top middle) Carnivora, kinkajou (*Potus flavos*), image by Kids Saving the Rainforest; (top right) Primates, Geoffroy's spider monkey (*Ateles geoffroyi*), image by Amanda Melin; (lower left) Didelphimorphia, woolly opossum (*Caluromus derbianus*), image by Christian Ziegler, reproduced with permission; (lower middle) Chiroptera, a Seba's short-tailed bat (*Carollia perspicillata*), image by Roberto Leonan Morim Novaes, reproduced with permission; (lower right) Scandentia, a pen-tailed treeshrew (*Ptilocercus lowii*), image shared with permission by Annette Zitzmann.

Frugivorous and nectarivorous diets have repeatedly emerged in mammal evolution and are found in many taxa, including rodents, bats, carnivores, primates and marsupials [23–26] (figure 1). Some species are of particular interest because of their higher reliance on floral nectars and/or fruits and, in turn, higher presumed dietary exposure to ethanol. For example, the pen-tailed treeshrew (*Ptilocercus lowii*) consumes fermented nectar in the wild without signs of motor impairment [12]. Bats are the second largest order of mammals with greater than 1400 species worldwide and are renowned for their ecological diversity [27]. In particular, the neotropical leaf-nosed bats (family Phyllostomidae) have varied diets, with species specializing on insects, blood (haematophagy), nectar and fruit [28]. Additional small mammals with omnivorous diets opportunistically feed on floral nectars and fruits, and may consume these resources in abundance when available [29,30]. Given this remarkable variation among closely related taxa, mammals are well suited for studying how dietary adaptations may relate to functional genetic variation across phylogenies of species with distinct life histories.

Here, we test the hypothesis that diverse frugivorous and nectarivorous mammalian species have convergently evolved a similar adaptation to metabolize ethanol through mutation in the *ADH7* gene. We predicted that primarily frugivorous and/or nectarivorous species will have substitutions at site 294 to valine or to other amino acids with similar physical and chemical properties, i.e. hydrophobic nature and a large side chain. Having a larger side chain on amino acid 294 makes the substrate binding pocket smaller. This lowers the K_m_—i.e. increases the enzyme affinity—for small alcohols like ethanol, while simultaneously disfavouring larger alcohols such as geraniol. Secondly, we predict that the *ADH7* genes of highly insectivorous, carnivorous or herbivorous species, which are less likely to be exposed to dietary ethanol, are pseudogenized through frameshift or mutations leading to premature stop codons due to relaxed selection. To test these predictions, we examined *ADH7* gene sequences of multiple groups of mammalian species including treeshrews, rodents, leaf-nosed bats and marsupials to study variation at site 294 and potential pseudogenes. Furthermore, we ran phylogenetic generalized linear models to test for statistically significant effects of frugivorous and/or nectarivorous diets on (i) variation at site 294 and/or (ii) putative *ADH7* gene functionality versus pseudogenization. Our research expands on data generated by recent study [15] by including *ADH7* sequences for an additional 89 ecologically relevant species, of which 59 are newly sequenced in this study, allowing us to better test adaptive hypotheses while controlling for phylogenetic effects.

## 2. Material and methods

### 2.1. Sample collection and DNA extraction

We generated new data from DNA derived from blood and tissue samples of 59 mammalian species: 27 bat, nine treeshrew, 11 rodent, four opossum and eight additional small to mid-sized mammal species in Central and South America, the Caribbean, as well as Southeast Asia. We extracted genomic DNA from all samples using the DNeasy Blood and Tissue Kit (Qiagen following the manufacturer's instructions. A list of sampled species, country of origin and DNA sequencing approach used is provided in table 1. Further details on sample collection, exportation and importation and permits are provided in the Ethics statement. All protocols were approved by the Life and Environmental Sciences Animal Care Committee at the University of Calgary (protocol nos. AC17-0094, AC-15-0161, AC-19-0167, AC-20-0052).

**Table 1.** The species included in this study and the country of origin for samples along with the sequencing approach used for the *ADH7* gene, i.e. target capture plus massive parallel sequencing, or Sanger sequencing following PCR amplification of exon 7.

order, family	species	common name	country of origin	target capture	Sanger
Carnivora, Mephitidae	*Conepatus semistriatus*	striped hog-nosed skunk	Costa Rica	**•**	
Carnivora, Procyonidae	*Nasua narica*	white-nosed coati	Costa Rica	**•**	
	*Potos flavus*	kinkajou	Costa Rica	**•**	
	*Procyon lotor*	raccoon	Costa Rica	**•**	
Chiroptera, Molossidae	*Molossus molossus*	velvety free-tailed bat	Dominica	**•**	
	*Tadarida brasiliensis*	Brazilian free-tailed bat	Dominica	**•**	
Chiroptera, Mormoopidae	*Pteronotus davyi*	Davy's naked-backed bat	Dominica	**•**	
Chiroptera, Phyllostomidae	*Anoura caudifer*	tailed tailless bat	Brazil	**•**	**•**
	*Ardops nichollsi*	lesser Antillean tree bat	Dominica	**•**	
	*Artibeus jamaicensis*	Jamaican fruit bat	Dominica	**•**	
	*Artibeus planirostris*	flat-faced fruit-eating bat	Brazil	**•**	**•**
	*Brachyphylla cavernarum*	Antillean fruit-eating bat	Dominica	**•**	
	*Carollia perspicillata*	Seba's short-tailed bat	Brazil	**•**	
	*Artibeus cinereus*	Gervais's fruit-eating bat	Brazil	**•**	**•**
	*Diaemus youngi*	white-winged vampire bat	Brazil		**•**
	*Diphylla ecaudata*	hairy-legged vampire bat	Brazil	**•**	**•**
	*Glossophaga soricina*	Pallas's long-tongued bat	Brazil	**•**	
	*Lonchophylla mordax*	Goldman's nectar bat	Brazil	**•**	**•**
	*Lophostoma brasiliense*	pygmy round-eared bat	Brazil	**•**	
	*Micronycteris sanborni*	Sanborn's big-eared bat	Brazil	**•**	**•**
	*Gardnerycteris crenulatum*	striped hairy-nosed bat	Brazil		**•**
	*Monophyllus plethodon*	lesser Antillean long-tongued bat	Dominica	**•**	
	*Platyrrhinus lineatus*	white-lined broad-nosed bat	Brazil	**•**	
	*Phyllostomus hastatus*	greater spear-nosed bat	Brazil	**•**	
	*Sturnira lilium*	little yellow-shouldered bat	Brazil	**•**	
	*Tonatia bidens*	greater round-eared bat	Brazil	**•**	**•**
	*Trachops cirrhosus*	fringe-lipped bat	Brazil	**•**	
Chiroptera, Pteropodidae	*Pteropus samoensis*	Samoa flying fox	Samoa	**•**	**•**
Chiroptera, Vespertilionidae	*Myotis dominicensis*	Dominican myotis	Dominica	**•**	
	*Myotis lavali*	LaVal's myotis	Brazil	**•**	**•**
	*Myotis levis*	yellowish myotis	Brazil	**•**	**•**
Didelphimorphia, Didelphidae	*Caluromys derbianus*	Derby's woolly opossum	Costa Rica	**•**	
	*Caluromys lanatus*	brown-eared woolly opossum	Peru	**•**	
	*Marmosa mexicana*	Mexican mouse opossum	Costa Rica	**•**	
	*Philander opossum*	gray four-eyed opossum	Costa Rica	**•**	
Eulipotyphla, Erinaceidae	*Echinosorex gymnura*	moonrat	Borneo	**•**	
Eulipotyphla, Soricidae	*Chimarrogale himalayica*	Himalayan water shrew	Borneo	**•**	
Pilosa, Bradypodidae	*Bradypus variegatus*	brown-throated sloth	Costa Rica	**•**	
Pilosa, Choloepodidae	*Choloepus hoffmanni*	Hoffmann's two-toed sloth	Costa Rica	**•**	
Rodentia, Cricetidae	*Nyctomys sumichrasti*	Sumichrast's vesper rat	Costa Rica	**•**	
	*Ototylomys phyllotis*	big-eared climbing rat	Costa Rica	**•**	
Rodentia, Dasyproctidae	*Dasyprocta punctata*	Central American agouti	Costa Rica	**•**	
Rodentia, Hystricidae	*Trichys fasciculata*	long-tailed porcupine	Borneo	**•**	
Rodentia, Muridae	*Leopoldamys sabanus*	long-tailed giant rat	Borneo	**•**	
Rodentia, Sciuridae	*Callosciurus notatus*	plantain squirrel	Borneo	**•**	
	*Lariscus hosei*	four-striped ground squirrel	Borneo	**•**	
	*Echinosciurus variegatoides*	variegated squirrel	Costa Rica	**•**	
	*Sundasciurus brookei*	Brooke's squirrel	Borneo	**•**	
	*Sundasciurus hippurus*	horse-tailed squirrel	Borneo	**•**	
	*Sundasciurus lowii*	Low's squirrel	Borneo	**•**	
Scandentia, Ptilocercidae	*Ptilocercus lowii*	pen-tailed treeshrew	Borneo		**•**
Scandentia, Tupaiidae	*Dendrogale melanura*	Bornean smooth-tailed treeshrew	Borneo	**•**	**•**
	*Dendrogale murina*	northern smooth-tailed treeshrew	Cambodia	**•**	
	*Urogale everetti*	Mindanao treeshrew	Philippines	**•**	**•**
	*Tupaia gracilis*	slender treeshrew	Borneo	**•**	
	*Tupaia longipes*	long-footed treeshrew	Borneo	**•**	**•**
	*Tupaia minor*	pygmy treeshrew	Borneo	**•**	**•**
	*Tupaia montana*	mountain treeshrew	Borneo	**•**	**•**
	*Tupaia tana*	large treeshrew	Borneo	**•**	**•**

### 2.2. Sequencing

We used two complementary approaches to examine *ADH7* sequence variation: (i) a targeted-capture massively parallel sequencing approach, and (ii) amplification of exon 7 followed by a Sanger sequencing approach. While the target capture approach allowed for the efficient investigation of most of the *ADH7* gene for a large number of species, Illumina sequencing has relatively high error rates and the effectiveness of the baits is not guaranteed for species without a close genomic reference available [31]. We therefore used Sanger sequencing for a subset of samples for which sufficient quantities of DNA were available, to validate the sequences obtained via massive parallel sequencing.

For the target capture approach, we used a custom set of biotinylated RNA probes (myBaits, Arbor Biosciences, Ann Arbor, MI) designed to capture the *ADH7* coding region in a wide range of mammals. Complementary RNA baits were designed using exons of *ADH7* coding sequences (mRNA) from species closely related to those of interest, which were retrieved from annotated genomes from the National Center for Biotechnology Information (NCBI) (electronic supplementary material, table S1). The RNA baits design used 3X tiling to give 35 938 baits following standard filtering criteria. Sequencing libraries were prepared by the University Core DNA (UCDNA) services at the University of Calgary with the NEBNext Ultra II FS Library Prep Kit (New England BioLabs). Following library preparation, we used complementary biotinylated RNA baits to capture *ADH7* gene sequences for species of interest, following the manufacturer's protocol. Hybridized library sequences were denatured, amplified and massive parallel sequenced on an Illumina NextSeq 500 using a 2 × 150 paired end NextSeq 500/550 mid-output v. 2.5 (300 Cycles) kit by the UCDNA services at the University of Calgary. For the second approach, we first amplified exon 7 of *ADH7* using custom-designed primers (electronic supplementary material, tables S2 and S3). We then purified and Sanger sequenced the product at the University of Calgary Centre for Health Genomics and Informatics using the same primers on an Applied Biosystems 3730xl (96 capillary) Genetic Analyzer using BigDye Terminator chemistry.

We find high concordance in the sequences generated by these two methods. For example, of the six treeshrew species for which we have both Sanger and Illumina sequences, exon 7 is 100% identical for four species and 99.24% identical for the remaining two species (one base difference in each case). In one species (*U. everetti)* a base was called as a C by Sanger sequencing and a T in the consensus sequence of the Illumina short-reads. Examining the short-reads shows support for both T/C bases at roughly 50% each, indicating a polymorphism in which different methods each picked up a different base. In the other case (*T. tana*) there is no evidence of a polymorphism, but the sequences are derived from two different individuals, so the one base difference could be due to individual variation. In both cases the variable bases are in the third position of a codon, so do not change the coding sequence.

### 2.3. Assembly and alignment

We removed adapters and trimmed Illumina short-reads using BBDuk from the BBTools suite [32]. We removed bases lower than *q* = 10 from the left and right ends of reads and only retained reads that were at least 25 bases long after trimming. We used the pipeline HybPiper (https://github.com/mossmatters/HybPiper/wiki) to extract and assemble gene sequences from the targeted enrichment sequencing reads [33]. Within HybPiper, sequence reads are mapped to reference gene sequences (such as those used to design the baits) with BWA sorted based on similarity, and then assembled into contigs on a gene-by-gene basis with SPAdes [34]. Exonerate is then used to align contigs against the reference gene sequences and extract coding regions. During analyses, we discovered the annotation of multiple *ADH7* genes in newer chromosome-level genome assemblies now available for some bat species [35]. The presence of a second *ADH7* gene in bats was not known at the time we designed the baits for our target capture sequencing, complicating our assembly pipeline. As expected with short-read sequencing data, attempts to fully assemble these paralogues separately from our reads were not successful; however, variation in bat *ADH7* sequences at site 294 could be determined by visually inspecting the mapped shorts reads in IGV [36].

To assemble reads generated via Sanger sequencing, forward and reverse reads were trimmed to remove bases with poor base call quality, and contigs were created with the software platform Geneious Prime (2021.1.1; https://www.geneious.com). Chromatograms were inspected for any polymorphic bases at site 294. We aligned all sequences newly generated for this study via Sanger and Illumina Sequencing, along with 85 *ADH7* sequences curated in a previous study [15] and 30 sequences newly mined from NCBI (electronic supplementary material, table S4) using MAFFT on the software platform Geneious Prime (2021.1.1) and cleaned manually. Stop codons found in sequences assembled via HybPiper were manually confirmed by inspecting the mapped short-reads with IGV [36] and only those confirmed by visual inspection were considered correct.

We retrieved some portion of *ADH7* for all 59 species newly sequenced. Of these, we were able to retrieve sequence data at the site of interest (294) for 55 species, excluding the hog-nosed skunk (*Conepatus semistriatus*), kinkajou (*Potus flavus*), moonrat (*Echinosorex gymnura*) and Himalayan water shrew (*Chimarrogale himalayica*). Through Sanger sequencing, we retrieved sequence data for exon 7 of *ADH7* for 19 out of 21 species, being unable to retrieve sequences with high base call quality for Gervais's fruit-eating bat (*Artibeus cinereus*) and the greater round-eared bat (*Tonatia bidens*).

### 2.4. Using phylogenetic generalized linear models to test for correlations with diet

**Figure 2.**
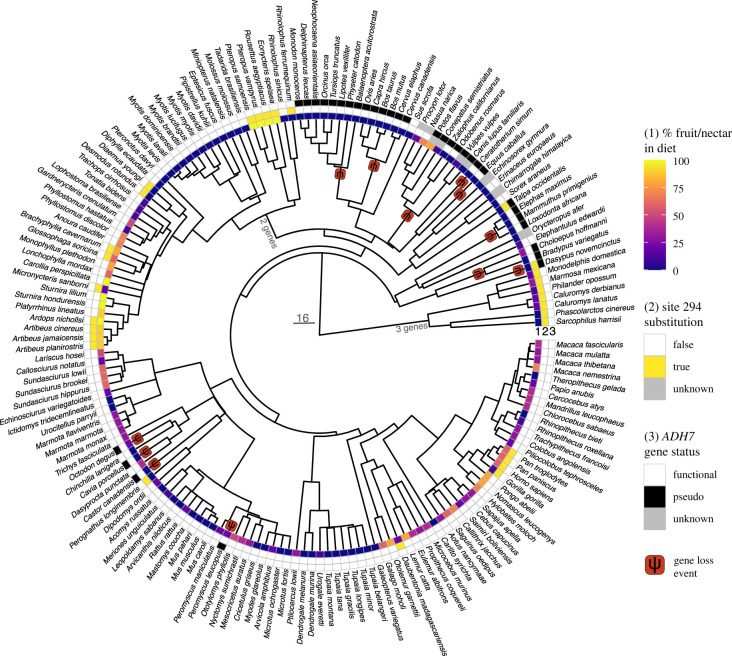
Evolutionary history of *ADH7* in mammals included in this study. Per cent fruit and/or nectar included in a species' diet is indicated in the first row of tip annotations. The second row indicates whether any substitutions away from the ancestral alanine are found at site 294 of *ADH7* and the third row indicates whether the gene is putatively functional or pseudogenized. Red boxes with the letter psi (ψ) indicate the inferred lineage in which gene loss events occurred, based on putatively shared loss-of-function mutations. Phylogeny via TimeTree ([41]; http://timetree.org), plotted with the R package ggtree [42].

To test if a frugivorous and/or nectarivorous diet correlates with (i) retention of a functional *ADH7* gene and/or (ii) substitutions to amino acids other than alanine at site 294 of the ADH class IV enzyme (encoded within exon 7 of *ADH7*) we ran phylogenetic generalized linear models (PGLMs). PGLMs are a modification of generalized linear models that consider the expected covariance structure of residuals, i.e. due to relatedness among species, to generate modified slope and intercept estimates that can account for autocorrelation due to phylogeny [37]. For each model we used the dietary proportions (specifically from per cent fruit and/or nectar in the diet) retrieved from EltonTraits 1.0 [38] as the predictor variable. We additionally ran the models with fruit and/or nectar in the diet as a binary predictor, and our results do not differ. (For sake of brevity, we report only the results generated by the former practice based on the per cent fruit/nectar in the diet.) To test our first hypothesis, we used a binary classification of the *ADH7* gene status (retention/pseudogenization; *n* = 166) as the dependent variable, and to test our second hypothesis we used the presence of a substitution at site 294 (yes/no) as a binary dependent variable (*n* = 133). For species with more than one *ADH7* gene, the species was included as having a substitution if at least one of the *ADH7* genes had an amino acid other than alanine at site 294. Because the majority of variation at site 294 was found within bats, we repeated the substitution analysis for bats only. We ran each PGLM using both the maximum penalized likelihood estimation (MPLE) and the IG10 methods, which differ slightly in how the calculated likelihood is penalized, following established protocols [15,39] using the function phyloglm within the R package phylolm [40]. Phylogenetic relationships among study species were based on TimeTree [41]. Missing data (‘unknown’, figure 2) were dropped prior to running the models.

## 3. Results

### 3.1. Diet and putative functional variation at site 294

We predicted that frugivorous and nectarivorous mammals would have substitutions at site 294 from the ancestral amino acid to a residue with high predicted affinity for ethanol. Alanine is the most likely candidate as the ancestral condition for mammals as it is present at site 294 in most species, including among early branching lineages. We found that two *ADH7* genes were annotated in several of the high-quality, long-read genome assemblies of multiple bat species (*Rhinolophus ferrumequinum*, *Rousettus aegyptiacus*, *Miniopterus natalensis*, *Phyllostomus hastatus*, *P. discolor*). Given the presence of two genes in multiple distantly related bat species, the most parsimonious inference is that *ADH7* was duplicated in the ancestor of bats. Among the bats examined (42 species), we found evidence of five amino acid variants at site 294 (figure 2, table 2). Valine was homozygously present in Pallas's long-tongued bat (*Glossophaga soricina*), the white-winged (*Diaemus youngi*) and common vampire bat (*Desmodus rotundus*), and the large flying fox (*Pteropus vampyrus*). The Samoa flying fox (*Pteropus samoensis*) and Egyptian fruit bat (*Rousettus aegyptiacus*) were homozygous for isoleucine at site 294. The greater horseshoe bat (*Rhinolophus ferrumequinum*), Sanborn's big-eared bat (*Micronycteris sanborni*), the tree bat *Ardops nichollsi*, flat-faced fruit-eating bat (*Artibeus planirostris*), Jamaican fruit bat (*Artibeus jamaicensis*) and Gervais's fruit-eating bat (*Artibeus cinereus*) were ‘heterozygous’ (i.e. either heterozygous, or possessing paralogues with different residues) with an alanine and a threonine at site 294. The Antillean fruit-eating bat (*Brachyphylla cavernarum*) had an alanine and glycine, respectively.

**Table 2.** Substitutions, or divergence in paralogues, at site 294. Genes are inferred to be functional unless noted as pseudogene. Twenty-eight out of 171 species examined were found to have substitutions at this site.

order	species	estimated % fruit and/or nectar	amino acid(s) at site 294
Chiroptera	*Rhinolophus ferrumequinum*	0	alanine/threonine
	*Eonycteris spelaea*	100	threonine
	*Rousettus aegyptiacus*	100	isoleucine
	*Pteropus vampyrus*	100	valine
	*Pteropus samoensis*	100	isoleucine
	*Desmodus rotundus*	0	valine
	*Diaemus youngi*	0	
	*Brachyphylla cavernarum*	80	alanine/glycine
	*Glossophaga soricina*	60	valine
	*Micronycteris sanborni*	20	alanine/threonine
	*Ardops nichollsi*	100	alanine/threonine
	*Artibeus cinereus*	90	
	*Artibeus jamaicensis*	90	
	*Artibeus planirostris*	90	
Rodentia	*Perognathus longimembris*	0	serine
Primates	*Daubentonia madagascariensis*	20	valine
	*Pan paniscus*	60	valine
	*Pan troglodytes*	60	
	*Homo sapiens*	60	
	*Gorilla gorilla*	10	
Dasyuromorphia	*Sarcophilus harrisii*	0	alanine/alanine/asparagine
Diprotodontia	*Phascolarctos cinereus*	0	valine/isoleucine/?
Didelphimorphia	*Monodelphis domestica*	20	alanine/aspartic acid/leucine
	*Marmosa mexicana*	30	
	*Philander opossum*	10	
	*Caluromys derbianus*	20	
	*Caluromys lanatus*	20	
Eulipotyphla	*Talpa occidentalis*	0	valine (pseudogene)

Marsupials have three paralogues of *ADH7*, as previously described [15]. All of the American marsupials (order Didelphimorphia) we investigated appear to follow the same pattern of having an alanine, aspartic acid and leucine in the three paralogues, respectively. (Coverage of the third gene in the brown-eared woolly opossum (*Caluromys lanatus*) was insufficient at site 294, but the closely related Derby's woolly opossum (*Caluromys derbianus* did follow this pattern). The koala (*Phascolarctos cinereus*) had a valine and isoleucine, but we could not conclusively identify the orthologous site in the third gene. In the Tasmanian devil (*Sarcophilus harrisii*) the pattern was alanine, alanine and asparagine, but we identified a premature stop codon in the second paralogue.

As previously described [16], African great apes (*Pan paniscus*, *Pan troglodytes*, *Homo sapiens* and *Gorilla gorilla*) and the aye-aye (*Daubentonia madagascariensis*) have a valine at site 294. The pen-tailed treeshrew (*Ptilocercus lowii*), all other treeshrews and almost all remaining placental mammals we sequenced or mined from publicly available data possess an alanine at site 294 (figure 2). The only exception was the little pocket mouse (*Perognathus longimembris*), which has serine at the critical site. This was the only species we found to possess this residue.

**Figure 3.**
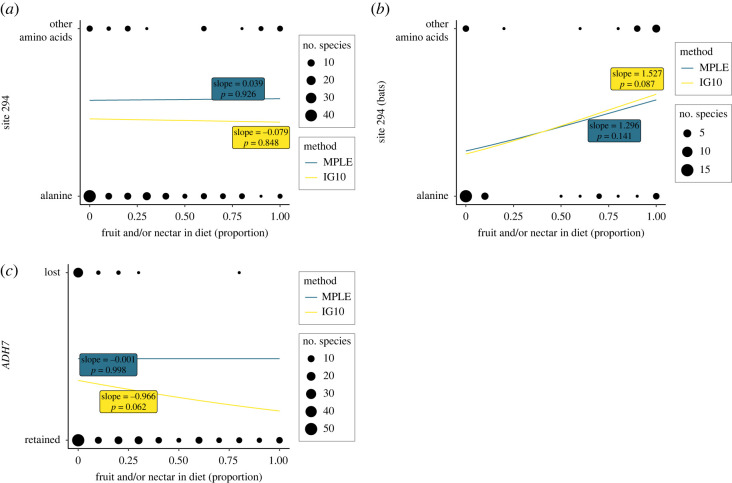
Results of the phylogenetic generalized linear models used to test if a frugivorous and/or nectarivorous diet correlates with amino acid substitutions at site 294 of the ADH class IV enzyme (encoded within exon 7 of *ADH7*) (*a*) in mammals overall and (*b*) in bats only, and (*c*) if a frugivorous and/or nectarivorous diet correlates with *ADH7* gene retention. For each prediction we ran two phylogenetic generalized linear models, the MPLE and the IG10 logistic regression. The slope and *p*-values for each method are in blue (for the MPLE method) and yellow (for the IG10 method) on each plot. Diet and genotypes of the species included are shown in black, with size of the dot indicating sample size.

We tested whether diet was a significant predictor of a substitution at site 294 of the ADH class IV enzyme(s) coded by *ADH7* using a phylogenetic generalized linear model with the IG10 and MPLE logistic regressions [43]. We found no evidence that the percentage of fruit and/or nectar in the diet predicts substitution frequencies at site 294 across mammals overall (IG10: slope = −0.079, *p*-value = 0.848; MPLE: slope = 0.039, *p*-value = 0.926; figure 3*a*), controlling for the impact of phylogenetic relationships among species.

When limiting the analysis to bats, the same patterns were found, although the slope of the relationship is suggestive (figure 3*b*) and raises the possibility of an underpowered result that should be treated with caution (IG10: slope = 1.527, *p*-value = 0.087; MPLE: slope = 1.296, *p*-value = 0.141).

### 3.2. Diet and putative gene functionality

We predicted that the *ADH7* genes of species with low levels of dietary ethanol exposure, i.e. highly insectivorous, carnivorous or herbivorous species, would be pseudogenized through frameshift or mutations leading to premature stop codons in the exons of *ADH7* due to relaxed selection. We found evidence of potential premature stop codons in 32 species, including species which had been previously described [15]. This included inferred pseudogenization events in the ancestral lineages of extant bovids, cetaceans, elephantids, carnivores and independently evolved premature stop codons in the nine-banded armadillo (*Dasypus novemcinctus*), white rhinoceros (*Ceratotherium simum*), horse (*Equus caballus*), common degu (*Octodon degus*), guinea pig (*Cavia porcellus*) and North American beaver (*Castor canadensis*). We newly identified premature stop codons in the assemblies of the Spanish mole (*Talpa occidentalis*) and white-footed mouse (*Peromyscus leucopus*), and in the brown-throated (*Dasypus variegatus*) and Hoffman's two-toed sloth (*Choloepus hoffmanni*) sequenced for this project (figure 2, electronic supplementary material, table S5). Coverage and/or completeness of the assembled gene was too low to determine the status of *ADH7* with confidence in four species, including the aardvark (*Orycteropus afer*), Himalayan water shrew (*Chimarrogale himalayaica*), moonrat (*Echinosorex gymnura*) and white-nosed coati (*Nasua narica*). The sequence assembled from the raccoon (*Procyon lotor*) sample was most similar to *Tupaia* and primates, casting doubt on the identity of the sample, and it was removed from downstream analyses.

We did not find a significant correlation between percentage of fruit and/or nectar in the diet and retention of a functional *ADH7* gene using the MPLE method (slope = −0.001, *p*-value = 0.998; figure 3*c*), nor the IG10 method (slope = −0.966, *p*-value = 0.062). As with the tests of site 294 variation in bats, the slope we generated for the IG10 model was not flat, so these tests might suffer from low power to detect an effect.

## 4. Discussion

Our aim was to evaluate the impact of diet on the evolution of a key gene in the ethanol metabolic pathway, *ADH7*, and to test the hypothesis that frugivorous and nectarivorous mammalian species have convergently evolved amino acid substitutions at functional site 294 that may enhance ethanol metabolic activity. Our findings are threefold: (i) most mammals possess alanine, the purported ancestral residue, at site 294 of the ADH class IV enzyme encoded by *ADH7* and we report many new cases of substitutions at this site, (ii) frugivorous bats are inferred to have undergone an *ADH7* gene duplication, and frugivorous, but not insectivorous species, had extensive variation at site 294 raising the possibility of adaptive variation, and (iii) we did not find a consistent, significant impact of the percentage of fruit/nectar in the diet and the presence of a substitution at site 294, or retention of a functional *ADH7* gene, although our confidence for rejecting this hypothesis is low in bats. We discuss these results in further detail, below.

### 4.1. Diet and putative functional variation at site 294

Within the ADH class IV enzyme encoded by gene *ADH7*, site 294 is well documented for its role in substrate binding, and plays a key role in predictive ethanol metabolic activity. Our first prediction was that species that are primarily frugivorous and/or nectarivorous would possess an amino acid at site 294 of *ADH7* that was likely to have high binding efficiency to ethanol, such as valine or another amino acid with similar properties, i.e. a relatively large side chain and hydrophobic nature [19]. This prediction was inspired by the observation that a substitution from alanine to valine in human *ADH7* leads to a 40-fold increase in ethanol metabolism [16].

We found that the majority of mammals in our study possess alanine at site 294, and this amino acid was reconstructed as the ancestral state. Interestingly, we also found a wide range of variation at this site, with evidence of phylogenetic clustering (figure 2). Frugivorous and nectarivorous bats possessed remarkable variation at site 294 of *ADH7* and annotations of newer chromosome-level long-read genome assemblies of species in this order show two paralogues of *ADH7*. This putative duplication is found in species across the chiropteran phylogeny, in both Yinpterochiroptera and Yangochiroptera, suggesting that it evolved in the ancestor of extant bats. The presence of a second *ADH7* gene in bats is a new discovery and was not known at the time we designed the baits for our target capture sequencing. Furthermore, short-read sequencing reads are often inadequate for the assembly of closely related and/or duplicated genes. This complicated our analysis pipeline and prevented full assembly of the *ADH7* genes from the targeted sequencing in bats. However, we were able to identify amino acid variation at site 294 from visual inspection of the mapped short-reads.

While the insectivorous bats nearly uniformly retained alanine (with one exception, discussed below), many frugivorous and nectarivorous bats had amino acid substitutions in one or both *ADH7* paralogues. We detected a minimum of nine independent transitions from alanine to other amino acids, and frugivorous/nectarivorous bats possessed four novel residues at site 294: valine, isoleucine, threonine, and glycine. In accordance with our predictions of convergence on valine, which increases ethanol metabolism 40-fold in primate *ADH7*, *Glossophaga soricina*, a nectarivorous phyllostomid species, and *Pteropus vampyrus*, a frugivore of the family Pteropodidae, each independently evolved valine at site 294. Intriguingly, Orbach *et al*. [44] found that the flying abilities of wild *Glossophaga soricina* are not affected after ingesting ethanol (1.5% ethanol concentration), suggesting that this species may be able to metabolize ethanol efficiently. The frugivorous Samoan flying fox (*Pteropus samoensis*) and Egyptian fruit bat (*Rousettus aegyptiacus*, also family Pteropodidae) both possessed an isoleucine substitution at site 294. Isoleucine, like valine, is a non-polar amino acid and possesses a larger side chain than alanine, suggesting that its presence at the critical site would increase the ethanol metabolic efficiency. It is possible the last common ancestor of the genera *Pteropus* and *Rousettus* possessed the substitution to isoleucine, and that an additional shift later occurred in *P. vampyrus*. Finally, the Antillean fruit-eating bat (*Brachyphylla cavernarum*) has evolved a glycine in one paralogue, and three bat lineages have independently evolved a threonine at site 294 in one or both paralogues. Unlike the other amino acids discussed, it is unclear how threonine or glycine might impact the enzyme's ability to bind ethanol or interact with the NAD + cofactor that binds nearby [20]; functional reconstitution and expression studies might shed further light on this question.

Considering other mammals, we replicated the finding of a valine substitution in humans and African great apes, and independently in aye-ayes, which are discussed in detail elsewhere [16,22]. Aye-ayes are best described as omnivorous, but are known to have a close relationship with the ethanolic nectar of the ravenala tree ([21]; Melin, Moritz, Guthrie, Johnson and Dominy 2017, unpublished data), suggesting that if diet selects for *ADH7* substitutions, gross dietary classifications may be missing important nuances. As previously identified in other marsupials (koala, Tasmanian devil and gray short-tailed opossum), we found that all American opossum species in our sample had three *ADH7* genes, which appear putatively functional: Derby's woolly opossum (*Caluromys derbianus*), gray short-tailed opossum (*Monodelphis domesticus*) and the gray four-eyed opossum (*Philander opossum*) all possessed alanine, aspartic acid and leucine at site 294 across the paralogues. Leucine is non-polar and has a similar-sized sidechain to isoleucine; its presence at the critical site probably leads to a relatively high binding affinity for ethanol. The functional variation of *ADH7* in marsupials is unclear, and presents a promising avenue for future study.

Several of our results were not consistent with our first prediction. Two of three vampire bats, *Diaemus youngi* and *Desmodus rotundus*, possessed a valine at site 294, while the hairy-legged vampire bat (*Diphylla ecaudata*) retained an alanine. Given the dietary specialization on blood, it is possible that the *ADH7* gene may be under relaxed selection in this lineage, or that other selective pressures are operating on it. For example, *ADH* genes have also been suggested to play roles in detoxification of other substances [45,46]. Finally, the retention of alanine in the pen-tailed treeshrew (*Ptilocercus lowii*), was surprising, as this species is renowned for consuming ethanol [12]. Given the behavioural and isotopic evidence of high metabolic activity for ethanol in treeshrews [12], it is possible that alternative molecular adaptations for metabolizing ethanol are in use, such as other alcohol dehydrogenases, aldehyde dehydrogenases, and enzymes in the microsomal ethanol oxidizing system (MEOS), catalase and non-oxidative pathways. Intriguingly, the glucuronidation (a non-oxidative) pathway has been hypothesized to contribute to ethanol metabolism in treeshrews [12,47] and might be worth future scrutiny to examine signs of adaptive evolution.

### 4.2. Diet and putative gene functionality

Our second prediction was that the *ADH7* genes of highly insectivorous, carnivorous/sanguinivorous, or herbivorous species will be pseudogenized due to relaxed selection, as they are less likely to be exposed to dietary ethanol. Our prediction was partially supported. We identified one or more independently derived putative premature stop codons among many non-frugivorous species, including the herbivorous Hoffmann's two-toed sloth (*Choloepus hoffmanni*) and brown-throated sloth (*Bradypus variegatus*). These newly sequenced species add to the species previously identified by Janiak *et al*. [15] to have pseudogenes, including many herbivores (bovids, elephants, rodents), carnivores (order Carnivora, cetaceans) and the insectivorous nine-banded armadillo (*Dasypus novemcinctus*). However, the phylogenetic generalized linear model we ran on all available species suggests the percentage of fruit and/or nectar in the diet was not significantly negatively correlated with possessing a putative *ADH7* pseudogene, which suggests the biological effect is probably weak and other factors may be at play. Of the animals newly sequenced in our study with carnivorous evolutionary histories, we found the striped hog-nosed skunk (*Conepatus semistriatus*, omnivorous) and kinkajou (frugivorous) shared a stop codon also found in other carnivores, such as dogs, wolves and foxes. This suggests that shifts to a partially or majority frugivorous diet, following ancestry focused on meat eating, have not coincided with regain of *ADH7* function for ethanol metabolism. It is possible that other genes have undergone neofunctionalization for ethanol metabolism, but we cannot test that hypothesis with our current dataset.

## 5. Conclusion and future directions

We report extensive new amino acid variation at kinematically important sites in the *ADH7* gene of bats and marsupials of the Americas, and confirm previous reports of variation in primates. Furthermore, we found evidence of potential premature stop codons, primarily in species for which fruit and/or nectar consumption is low, indicating potential relaxation of selection when ethanol is not common in the diet. These results raise the intriguing possibility that some frugivorous/nectarivorous species are able to metabolize ethanol more efficiently than most mammals. While we did not find evidence that diet was a significant predictor of substitutions at site 294 or *ADH7* gene functionality, our statistical tests might be underpowered. Future studies of positive selection (e.g. *d*_N_/*d*_S_) examining the complete coding sequence of the entire gene may be fruitful. Furthermore, our measures of the importance of ethanol in the diet is crude, and ethanol could be more important in the diet of some species than others, including those classified as omnivores, where overall fruit and nectar in the diet might be moderate. Finally, data on enzyme kinetics that test the efficiency of ADH class IV enzymes to evaluate the impacts of isoleucine, leucine, valine, threonine, serine and glycine at the critical site 294, in the context of the species-specific amino acid composition (e.g. [16]), would provide functional insight. Detailed study of genes involved in other ethanol metabolic pathways and on the ethanol content of wild foods consumed by the species reported here (e.g. [7]), as well as more nuanced examination of omnivorous species which can consume a great deal of nectar or fruit [30], would be instructive and shed further light on this intriguing topic.

## Data Availability

Raw sequence reads are deposited in the SRA (BioProject PRJNA926128) and individual short-read alignment files (bam format) are available on DataDryad (https://doi.org/10.5061/dryad.t4b8gtj6r) [48]. Sanger sequencing trace files and all data used in the phylogenetic analyses and scripts to recreate the analyses in R are also provided in the DataDryad repository (https://doi.org/10.5061/dryad.t4b8gtj6r). The data are provided in electronic supplementary material [49].
